# A nomogram to predict conversion of laparoscopic surgery to laparotomy for Choledocholithiasis

**DOI:** 10.1186/s12893-023-02275-1

**Published:** 2023-12-08

**Authors:** Yitao Zheng, Haoyang Lv, Zhuoqun Lin, Hongqi Shi, Xiaming Huang

**Affiliations:** Department of Hepatobiliary Surgery, The First Affiliated Hospital, Wenzhou Medical University, Wenzhou, 325000 Zhejiang Province China

**Keywords:** Conversion, Laparoscopic surgery, Laparotomy, Nomogram, Choledocholithiasis

## Abstract

**Background:**

Laparoscopic surgery is effective for treating common bile duct (CBD) stones. However, it has high requirements for surgeons and the risk of conversion to laparotomy cannot be ignored. However, when conditions during surgery are not favorable, persisting with laparoscopic procedures blindly can lead to serious complications. Our study aimed to establish a nomogram model for predicting conversion of laparoscopic to laparotomy for choledocholithiasis.

**Materials and methods:**

A total of 867 patients who were diagnosed with choledocholithiasis and underwent laparoscopic surgery were randomly divided into a training group (70%, *n* = 607) and a validation group (30%, *n* = 260). A nomogram was constructed based on the results of logistic regression analysis. The area under the receiver operating characteristic curve (AUC), calibration curve, and decision curve analysis (DCA) were used to assess the predictive performance of the nomogram.

**Results:**

Previous upper abdominal surgery, maximum diameter of stone ≥12 mm, medial wall of the duodenum stone, thickening of the gallbladder wall, thickening of CBD wall, stone size/CBD size ≥0.75, and simultaneous laparoscopic hepatectomy were included in the nomogram. The AUC values were 0.813 (95% CI: 0.766–0.861) and 0.804 (95% CI: 0.737–0.871) in the training and validation groups, respectively. The calibration curve showed excellent consistency between the nomogram predictions and actual observations. DCA showed a positive net benefit for the nomogram.

**Conclusions:**

We constructed a nomogram with a good ability to predict conversion to open surgery in laparoscopic surgery for choledocholithiasis, which can help surgeons to make a reasonable operation plan before surgery and timely convert to laparotomy during operation to reduce potential harm to the patient.

**Supplementary Information:**

The online version contains supplementary material available at 10.1186/s12893-023-02275-1.

## Introduction

Approximately 3–18% of patients with gallbladder stones have secondary choledocholithiasis [1, 2]. Previously, laparotomy was the gold standard treatment for choledocholithiasis, however, this procedure often required large abdominal incisions. Postoperative incision infections and pain are common in these patients, resulting in longer hospital stays [3]. With the development of laparoscopic technology, laparoscopic common bile duct exploration (LCBDE) surgery is favored by an increasing number of surgeons because this procedure is less invasive than open surgery, with a lower risk of infection and faster recovery for the patients [4]. Patients with common bile duct (CBD) stones sometimes also have intrahepatic bile duct (IHD) stones [1]. Laparoscopic hepatectomy (LH) has been shown to be safe for some patients with IHD stones [5, 6]. Therefore, for patients with concomitant intrahepatic and extrahepatic bile duct stones, simultaneous LCBDE and LH procedures are required [7, 8].

LCBDE and LH require proficiency in laparoscopic hepatobiliary system anatomy, skilled laparoscopic suturing and knot-tying, and endoscopic techniques [9]. Even when performed by experienced surgeons, there is a 5% probability of conversion to open surgery [10]. Conversion is neither a surgical complication nor a surgical failure. Rather, it is a change in the surgical approach. Blind continuation of laparoscopic surgery may lead to serious complications when circumstances make it inappropriate to continue [11]. Therefore, a scoring system is urgently needed to help surgeons choose between a more rational preoperative and intraoperative surgical approach in laparoscopic surgery for choledocholithiasis.

Our study aimed to establish a nomogram to evaluate the risk of conversion to open surgery in patients undergoing laparoscopic surgery for choledocholithiasis, thereby enabling surgeons to judge the complications of the procedure and reduce patient trauma.

## Materials and methods

### Patients

We collected medical records of patients who were diagnosed with choledocholithiasis with or without hepatolithiasis and underwent laparoscopic surgery from January 2015 to September 2020 at the First Affiliated Hospital of Wenzhou Medical University. The exclusion criteria were as follows: (1) lack of preoperative clinical data, (2) intraoperative or postoperative diagnosis of gallbladder, bile duct, and ampullary tumors, (3) congenital anomalies of the hepatobiliary system, (4) Mirizzi Syndrome.

The First Affiliated Hospital of Wenzhou Medical University’s Ethics Committee approved this study (No.:Y(2022).R194). Due to the retrospective nature of this study and the anonymized data of all patients, the Ethics Committee of the First Affiliated Hospital of Wenzhou Medical University waived the requirement for informed consent.

### Surgical procedures

**LCBDE:** all patients underwent surgery by either associate chief surgeons or chief surgeons with more experience. After a successful anesthesia, the patient is placed in a supine position. Standard disinfection measures are followed and a 10 mm arc-shaped incision is made at the lower edge of the navel. The pneumoperitoneum needle is then successfully inserted into the abdominal cavity, injecting carbon dioxide to establish 15 mmHg of pneumoperitoneum. A 10 mm diameter Trocar is punctured into the abdominal cavity, followed by the placement of a laparoscope. The patient’s position is then adjusted to a semi-fowler’s with the right side slightly elevated. Based on the location of the liver and gallbladder, an approximately 10 mm long incision is made beneath the xiphoid process on the right side, along with two other incisions, each about 5 mm long, below the costal margin on the right mid-clavicular line and the right mid-axillary line respectively. Corresponding Trocars with diameters of 10 mm and 5 mm are punctured into the abdominal cavity. Under laparoscopy, the common bile duct is exposed. A longitudinal incision of about 1.5 cm is made on the upper segment of the common bile duct to the duodenum. Stones are initially removed using stone forceps, followed by inspection with a choledochoscope. Sterile saline washing, stone retrieval baskets, and holmium laser lithotripsy are among the methods used for stone removal until no stones are detected under the choledochoscope.

**LH:** after anesthesia, the patient is positioned in the supine position. The four-trocar technique is commonly used in most cases. As described above, a trocar is placed below the umbilicus to establish pneumoperitoneum, maintaining an intra-abdominal pressure of 12–15 mmHg. Additionally, a 5 mm trocar is placed below the costal margin along the midclavicular line on the right side and another along the right mid-axillary line. A 12 mm trocar is positioned below the xiphoid process. The liver parenchyma is excised using an ultrasonic scalpel, in conjunction with instruments like electrocautery, Hem-o-lock clips, and the Endo-Line Cutter. The falciform and coronary ligaments are severed with the ultrasonic scalpel. Careful dissection of the arteries and veins in the left lateral segment is performed, followed by clamping with Hem-o-lock clips, and then separation. The left hepatic vein is transected from the second hepatic portal, using clamps to separate it. The liver parenchyma is then transected transversely with an endolinear cutter. The excised specimen is placed into a specimen bag, crushed, and extracted through a 12 mm port. The liver’s cut surface is meticulously inspected to ensure there is no bleeding or bile leak, followed by thorough lavage of the abdominal cavity. A drainage tube is placed as per routine. A T-tube is usually placed postoperatively. The method for exploring the common bile duct and performing a cholecystectomy is the same as described.

### Data collection

The demographic and clinical data, including age, sex, body mass index (BMI), diabetes, hypertension, previous upper abdominal surgery (PUAS), history of endoscopic retrograde cholangiopancreatography (ERCP), and pancreatitis were recorded. Laboratory blood test results included white blood cell count, albumin (ALB), total bilirubin, alkaline phosphatase, alanine aminotransferase, aspartate aminotransferase, and gamma-glutamyl transpeptidase levels. Preoperative abdominal ultrasonography, computed tomography, magnetic resonance imaging, or magnetic resonance cholangiopancreatography was evaluated by an experienced radiologist. The following data were collected: the maximum diameter of CBD stone, CBD stone’s location, The number of stones in CBD, IHD stone, gallbladder wall thickness, CBD wall thickness, the maximum diameter of CBD, and stone size/CBD size. All the included data were the most recent preoperative results for the patients. In addition, the type of surgery performed was also recorded.

Using the duodenum and pancreas as the boundary, we divided the stone locations into four parts: duodenal upper segment, duodenal posterior segment, pancreatic segment, and medial wall of the duodenum [12, 13]. Some patients may have stones at more than one site simultaneously. The maximum diameter of CBD stone was defined as the transverse diameter perpendicular to the wall of the bile duct. The wall of the gallbladder was considered thickened if it was > 4 mm [14]. CBD wall thickness > 2 mm was defined as CBD wall thickening [15]. Patients with previous cholecystectomy were considered to have a gallbladder wall thickness of ≤4 mm and to have a history of PUAS. All patients underwent LCBDE surgery, and some had undergone laparoscopic cholecystectomy (LC), LH, or holmium laser lithotripsy simultaneously.

We concurrently computed the ratio between the maximum diameter of the bile duct stones and the maximum diameter of the common bile duct for each patient. By calculating the Youden’s Index, we found that the optimal cut-off value is 0.75.

In addition, we recorded the operative time, stone clearance rate, T-tube placement, mortality, incision infection, postoperative complications, duration of postoperative antibiotic use, and postoperative hospital stay to analyze the effect of conversion to laparotomy during the perioperative period.

### Statistical analysis

IBM SPSS 23.0 (IBM USA) was used to process the data. According to the commonly used clinical cut-off points based on the relevant literature, some measurements in the original data were classified and transformed into count data, and the numerical values and percentages were used for statistical description. Normally distributed variables were expressed as mean ± standard deviation, and differences between groups were analyzed using the t-test. Variables with skewed distributions were expressed as medians (25th percentile, 75th percentile), and the Mann-Whitney test was used to assess differences between the two groups. Categorical variables were summarized as numbers and frequencies, and univariate analyses were performed using Fisher’s exact test or Pearson’s chi-square test. Univariate analysis was performed using the chi-square test or Fisher exact probability method to screen the relative risk factors. Multivariate logistic regression analysis (stepwise forward conditional) was used to screen the independent risk factors. All patients were randomly divided into a training group and a validation group in a 7:3 ratio. Multifactorial logistic regression analysis was conducted on the patients in the training group to construct a nomogram. The validation group was then used to assess the predictive performance of the nomogram. The “rms” package (R version 4.2.1) was used to establish the nomogram, and then bootstrap repeated sampling was used for internal verification and calibration curve drawing. To reduce overfitting bias, the nomogram model was internally verified using 1000 repeated samplings. The area under the receiver operating characteristic (ROC) curve (AUC) was used to evaluate model discrimination. Decision curve analysis (DCA) was used to quantify the net benefit under different threshold probabilities in the patient cohort, which can guide clinical decision-making. Across all tests, statistical significance was set at *P* < 0.05.

## Results

### Basic information

**Fig. 1:**
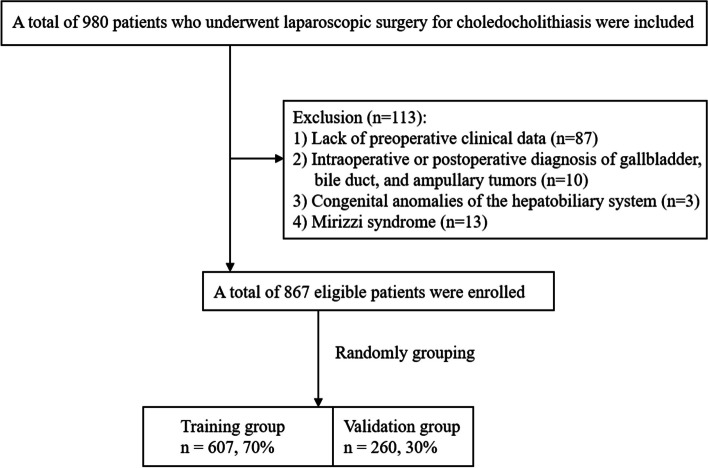
Flowchart showing patient selection process

Figure 1 shows the flow chart for patient selection. A total of 867 patients were enrolled in the study cohort, of which 607 patients were assigned to the training group and the remaining 260 to the validation group. There were no significant differences in baseline characteristics between the two groups (*P* > 0.05) (Table 1). A total of 151 patients underwent conversion to open surgery. The reasons for conversion to laparotomy were as follows: (1) Anatomical difficulties in the gallbladder triangle(25.2%), (2) severe abdominal adhesions (27.8%), (3) difficulty in stone removal (23.2%), (4) excessive number of stones (4.6%), (5) difficulties stopping bleeding (8.6%), (6) bile duct duodenal fistula or gallbladder duodenal fistula (8.6%), and (7) severe liver atrophy (2.0%).

**Table 1:** Baseline characteristics of training and validation groups

Variables	Whole group (*n* = 867)	Training group (*n* = 607)	Validation group (*n* = 260)	*P* value
Age [year]				0.051
≤ 60	347(40.0)	240(39.5)	107(41.2)	
60 ~ 80	459(52.9)	332(54.7)	127(48.8)	
>80	61(7.0)	35(5.8)	26(10.0)	
Sex				0.910
Male	386(44.5)	271(44.6)	115(44.2)	
Female	481(55.5)	336(55.4)	145(55.8)	
BMI [Kg/m^2^]				0.545
< 24	565(65.2)	389(64.1)	176(67.7)	
24 ~ 28	249(28.7)	181(29.8)	68(26.2)	
> 28	53(6.1)	37(6.1)	16(6.2)	
Diabetes	114(13.1)	85(14.0)	29(11.2)	0.274
Hypertension	265(30.6)	183(30.1)	82(31.5)	0.684
PUAS	147(17.0)	103(17.0)	44(16.9)	> 0.95
History of ERCP	45(5.2)	29(4.8)	16(6.2)	0.406
Pancreatitis	55(6.3)	33(5.4)	22(8.5)	0.097
WBC[*10^9^/L]				0.370
≤ 10	787(90.8)	547(90.1)	240(92.3)	
> 10	80(9.2)	60(9.9)	20(7.7)	
ALB [g/L]				0.428
≤ 35	231(26.6)	157(25.9)	74(28.5)	
> 35	636(73.4)	450(74.1)	186(71.5)	
TBIL [μmol/L]				0.683
≤ 17.1	411(46.1)	285(47.0)	126(48.5)	
> 17.1	456(53.9)	322(53.0)	134(51.5)	
ALP [U/L]				0.421
≤ 125	260(30.0)	187(30.8)	73(28.1)	
> 125	607(70.0)	420(69.2)	187(71.9)	
ALT [U/L]				0.421
≤ 40	243(28.0)	175(28.8)	68(26.2)	
> 40	624(72.0)	432(71.2)	192(73.8)	
AST [U/L]				0.582
≤ 40	312(36.0)	222(36.6)	90(34.6)	
> 40	555(64.0)	385(63.4)	170(65.4)	
GGT [U/L]				0.374
≤ 50	138(15.9)	101(16.6)	37(14.2)	
> 50	729(84.1)	506(83.4)	223(85.8)	
Maximum diameter of CBD stone [mm]				0.518
< 12	563(64.9)	390(64.3)	173(66.5)	
≥ 12	304(35.1)	217(35.7)	87(33.5)	
CBD stone’s location				
Duodenal upper segment				0.620
Yes	181(20.9)	124(20.4)	57(21.9)	
No	686(79.1)	483(79.6)	203(78.1)	
Duodenal posterior segment				0.470
Yes	331(38.2)	227(37.4)	104(40.0)	
No	536(61.8)	380(62.6)	156(60.0)	
Pancreatic segment				0.566
Yes	635(73.2)	448(73.8)	187(71.9)	
No	232(26.8)	159(26.2)	73(28.1)	
Medial wall of the duodenum				0.887
Yes	62(7.2)	43(7.1)	19(7.3)	
No	824(92.8)	564(92.9)	241(92.7)	
The number of stones in CBD				0.880
Negative/sludge	33(3.8)	24(4.0)	9(3.5)	
Single	384(44.3)	266(43.8)	118(45.4)	
Multiple	450(51.9)	317(52.2)	133(51.2)	
IHD stone				0.466
Yes	156(18.0)	113(18.6)	43(16.5)	
No	711(82.0)	494(81.4)	217(83.5)	
Thickening of gallbladder wall	321(37.0)	230(37.9)	91(35.0)	0.419
Thickening of CBD wall	63(7.3)	44(7.2)	19(7.3)	> 0.95
Diameter of CBD	13.3(10.7,17.0)	13.9(11.0,17.0)	13.0(10.0,16.1)	0.120
Stone size/CBD size				0.190
< 0.75	424(48.9)	288(47.4)	136(52.3)	
≥ 0.75	443(51.1)	319(52.6)	124(47.7)	
Surgery method				
Conversion to laparotomy	151(17.4)	102(16.8)	49(18.8)	0.468
Simultaneous LC				0.442
Yes	754(87.0)	524(86.3)	230(88.5)	
No	113(13.0)	83(13.7)	30(11.5)	
Simultaneous LH				0.426
Yes	91(10.5)	67(11.0)	24(9.2)	
No	776(89.5)	540(89.0)	236(90.8)	
Laser lithotripsy	19(2.2)	12(2.0)	7(2.7)	0.613

Data are expressed as *n* (%) unless otherwise specified

*BMI* body mass index, *PUAS* previous upper abdominal surgery, *ERCP* endoscopic retrograde cholangiopancreatography, *WBC* white blood cell, *ALB* albumin total bilirubin, *TBIL* total bilirubin, *ALP* alkaline phosphatase, *ALT* alanine aminotransferase, *AST* aspartate aminotransferase, *GGT* gamma-glutamyl transpeptidase, *CBD* common bile duct, *IHD* intrahepatic bile duct, *LC* laparoscopic cholecystectomy, *LH* laparoscopic hepatectomy

In the entire study population, the average age of the participants was 61.8 ± 14.4 years. Most CBD stones were located in the duodenal posterior and pancreatic segment, and 450 patients (51.9%) had two or more stones.

### Univariate and multivariate analysis

Table 2 shows the univariate and multivariate logistic regression analysis of factors influencing conversion to laparotomy. The following 11 factors were included in the univariate logistic regression: Female, PUAS, ALB< 35 g/L, maximum diameter of stone ≥12 mm, medial wall of the duodenum stone, IHD stone, thickening of the gallbladder wall, thickening of CBD wall, diameter of CBD, stone size/CBD size ≥0.75, and simultaneous LH. Finally, in the multivariate logistic regression, the following seven factors were identified as independent risk factors for predicting conversion to laparotomy: PUAS, maximum diameter of stone ≥12 mm, medial wall of the duodenum stone, thickening of the gallbladder wall, thickening of CBD wall, stone size/CBD size ≥0.75 and simultaneous LH.

**Table 2:** Univariate and multivariate analyses of each factor’s ability in predicting conversion

Characteristic	Univariable analysis		Multivariable analysis	
	OR (95% CI)	*P* value	OR (95% CI)	*P* value
Age [year]		0.276	–	
≤ 60	Ref			
60 ~ 80	1.175(0.745–1.851)			
>80	1.962(0.850–4.529)			
Sex		0.040^*^	–	0.128
Male	Ref			
Female	1.566(1.021–2.401)			
BMI [Kg/m^2^]		0.551	–	
< 24	Ref			
24 ~ 28	1.014(0.638–1.611)			
> 28	0.270(0.063–1.149)			
Diabetes (Yes/No)	0.789(0.411–1.514)	0.476	–	
Hypertension (Yes/No)	0.758(0.468–1.230)	0.262	–	
PUAS (Yes/No)	2.314(1.409–3.799)	0.001^*^	4.014(2.077–7.755)	< 0.001^*^
History of ERCP surgery (Yes/No)	1.310(0.519–3.302)	0.567	–	
Pancreatitis (Yes/No)	0.306(0.072–1.299)	0.108		
WBC[*10^9^/L]		0.739	–	
≤ 10	Ref			
> 10	1.125(0.563–2.246)			
ALB [g/L]		0.018^*^	–	0.477
≤ 35	Ref			
> 35	0.578(0.367–0.910)			
TBIL [μmol/L]		0.267	–	
≤ 17.1	Ref			
> 17.1	0.786(0.513–1.203)			
ALP [U/L]		0.921	–	
≤ 125	Ref			
> 125	1.024(0.645–1.625)			
ALT [U/L]		0.922	–	
≤ 40	Ref			
> 40	1.024(0.639–1.640)			
AST [U/L]		0.769	–	
≤ 40	Ref			
> 40	1.069(0.685–1.667)			
GGT [U/L]		> 0.95	–	
≤ 50	Ref			
> 50	0.998(0.564–1.766)			
Maximum diameter of CBD stone [mm]		< 0.001*		0.003^*^
< 12	Ref		Ref	
≥ 12	2.635(1.705–4.073)		2.312(1.318–4.057)	
CBD stone’s location				
Duodenal upper segment (Yes/No)	1.612(0.989–2.627)	0.055	–	
Duodenal posterior segment (Yes/No)	1.044(0.673–1.618)	0.848	–	
Pancreatic segment (Yes/No)	0.873(0.543–1.402)	0.573	–	
Medial wall of the duodenum (Yes/No)	4.588(2.406–8.748)	< 0.001^*^	5.393(2.513–11.574)	< 0.001^*^
The number of stones in CBD		0.711	–	
Negative/sludge	Ref			
Single	0.911(0.296–2.803)			
Multiple	1.096(0.361–3.330)			
IHD stone (Yes/No)	1.991(1.220–3.249)	0.006^*^	–	0.182
Thickening of gallbladder wall (Yes/No)	2.316(1505–3.564)	< 0.001^*^	4.318(2.454–7.597)	< 0.001^*^
Thickening of CBD wall (Yes/No)	6.037(3.195–11.411)	< 0.001^*^	4.513(2.111–9.646)	< 0.001^*^
Diameter of CBD	1.055(1.019–1.093)	0.002^*^	–	0.153
Stone size/CBD size		< 0.001^*^		0.026^*^
< 0.75	Ref		Ref	
≥ 0.75	2.650(1.666–4.216)		1.909(1.078–3.378)	
Surgery method				
Simultaneous LC (Yes/No)	0.689(0.389–1.221)	0.202	–	
Simultaneous LH (Yes/No)	4.521(2.624–7.789)	< 0.001^*^	7.032(3.683–13.426)	< 0.001^*^
Laser lithotripsy (Yes/No)	2.536(0.749–8.586)	0.135		

*BMI* body mass index, *PUAS* previous upper abdominal surgery, *ERCP* endoscopic retrograde cholangiopancreatography, *WBC* white blood cell, *ALB* albumin total bilirubin, *TBIL* total bilirubin, *ALP* alkaline phosphatase, *ALT* alanine aminotransferase, *AST* aspartate aminotransferase, *GGT* gamma-glutamyl transpeptidase, *CBD* common bile duct, *IHD* intrahepatic bile duct, *LC* laparoscopic cholecystectomy, *LH* laparoscopic hepatectomy, *OR* odds ratio, *CI* confidence interval

* *P* value < 0.05

### Construction of the nomogram

**Fig. 2:**
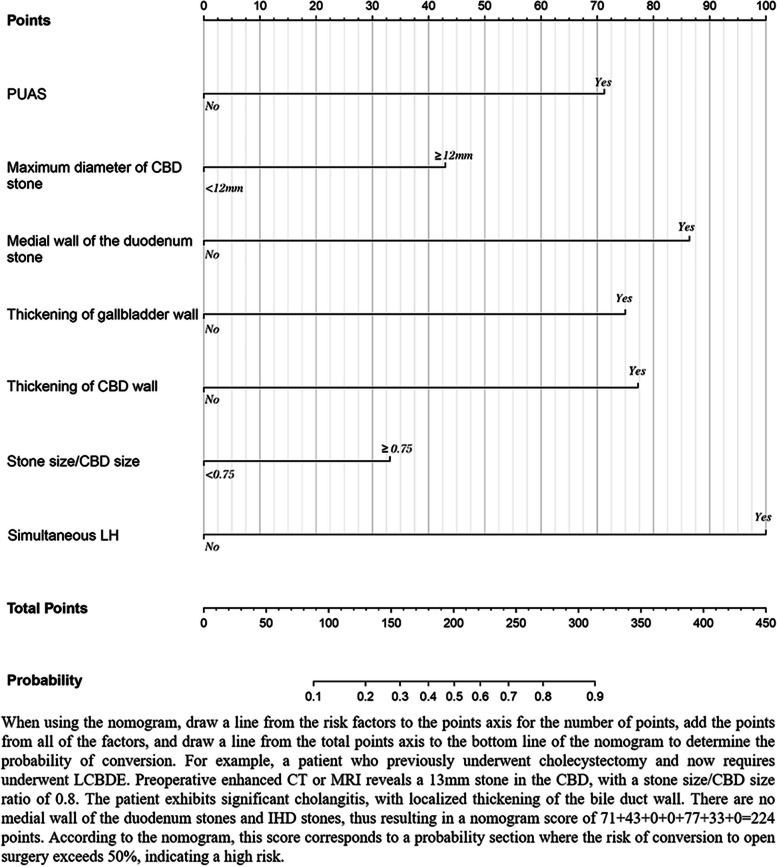
Nomogram for predicting the risk of conversion to open surgery in laparoscopic surgery for choledocholithiasis. PUAS, previous upper abdominal surgery; CBD, common bile duct; LH, laparoscopic hepatectomy; IHD, intrahepatic bile duct

Combined with the above seven independent risk factors, a prediction model was constructed and presented in the form of a nomogram (Fig. 2). The scores included in the nomogram prediction model for PUAS, maximum diameter of stone ≥12 mm, medial wall of the duodenum stone, thickening of the gallbladder wall, thickening of CBD wall, stone size/CBD size ≥0.75 and simultaneous LH were 71, 43, 86, 75, 77, 33 and 100, respectively. The nomogram was scored from 0 to 450 points. We also calculated the total score of each patient and based on that, the Youden-derived optimal cut-off value for the nomogram was 119 points, the nomogram had a sensitivity of 72.5% and specificity of 79.4% at this threshold. Using this score as the boundary, we assigned patients below this score as low-risk patients and those above this score as high-risk patients.

**Fig. 3:**
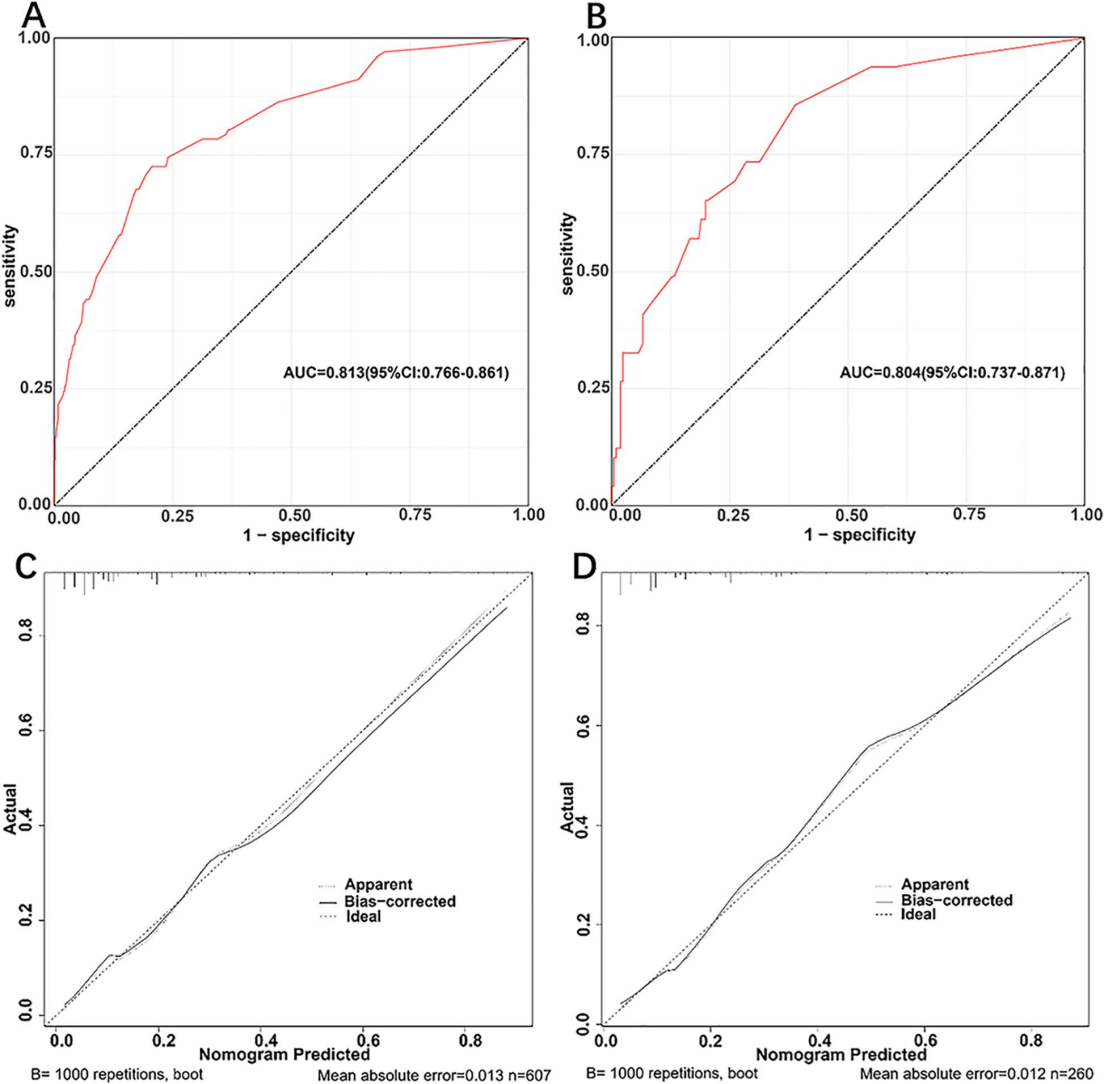
Discrimination and validation of the nomogram. **A** and **B** ROC curves of the nomogram for the training and validation groups, respectively. **C** and **D** Calibration plots for the nomogram in the training and validation groups, respectively. In the calibration plot, the x-and y-axis represents the nomogram-predicted and the actual probabilities, respectively. ROC, receiver operating characteristic; AUC, area under curve; CI, confidence interval

**Fig. 4:**
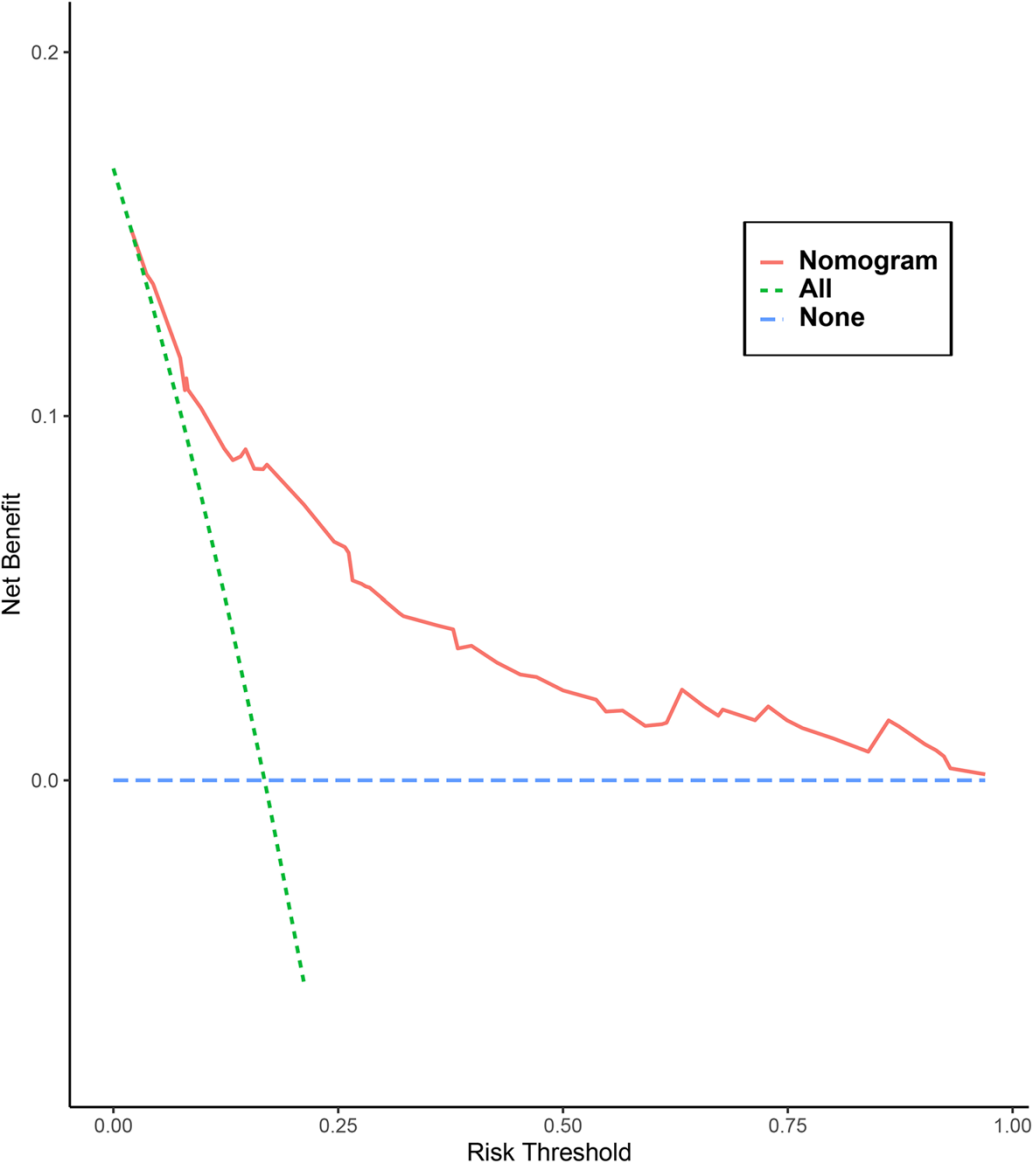
DCA curves for the nomogram in the training group. The x-axis represents the threshold probabilities and the y-axis measures the net benefit. The red line represents the nomogram. The dotted blue line represents that all patients underwent the conversion to laparotomy surgery. The dotted green line represents that all patients underwent laparoscopic surgery without conversion. The net benefit is calculated by adding the true positives and subtracting the false positives. DCA, decision curve analysis

The AUCs for the prediction nomogram was 0.813 (95% CI: 0.766–0.861) in the training (Fig. 3A) and 0.804 (95% CI: 0.737–0.871) in the validation group (Fig. 3B). The calibration curves of the training (Fig. 3C) and validation group (Fig. 3D) indicated the good consistency between the observed and predicted values, with the *P*-value for the Hosmer-Lemeshow test of 0.202 and 0.109, respectively. Furthermore, the nomogram-related DCA curves in the training (Fig. 4) showed good clinical application ability, suggesting a preferable positive net benefit.

### Perioperative outcomes

Table 3 shows the perioperative outcomes of the conversion and non-conversion groups. The operative time, duration of postoperative antibiotic use, incidence of incision infection, and length of postoperative hospital stay were higher in the conversion group than in the non-conversion group. There were no significant differences in stone clearance rate, postoperative mortality, or complications between the two groups. More patients in the conversion group underwent T-tube placement during the operation.

**Table 3:** Perioperative outcomes between the conversion and non-conversion groups

Perioperative outcomes	Conversion group (*n* = 151)	Non-conversion group (*n* = 716)	*P* value
Operative time [min]	160(130,200)	100(80,120)	< 0.001^*^
Stone clearance			0.092
Yes	142(94.0)	694(96.9)	
No	9(6.0)	22(3.1)	
T-tube placement			< 0.001^*^
Yes	139(92.1)	399(55.7)	
No	12(7.9)	(44.3)	
Mortality			> 0.95
Yes	0(0%)	1(0.1%)	
No	151(100%)	715(99.9%)	
Duration of Postoperative Antibiotics (day)	10(8,13)	7(6,9)	< 0.001^*^
Incision infection			< 0.001^*^
Yes	16(10.6)	8(1.1%)	
No	135(89.4)	708(98.9)	
Postoperative complications			0.536
Yes	9(6.0)	34(4.7)	
No	142(94.0)	682(95.3)	
Postoperative hospital stay (day)	11(9,16)	8(7,10)	< 0.001^*^

Data are expressed as *n* (%) unless otherwise specified

^*^P value < 0.05

## Discussion

Accurate preoperative stratification and intraoperative conversion to open surgery are key factors for controlling the quality of laparoscopic surgery and patient safety [16]. Previous studies [10, 17] have suggested that CBD edema, maximum stone diameter, multiple stones, and impacted stones are factors for conversion in LCBDE. However, they only predicted difficult factors without validation, and their clinical data were relatively limited. The seven independent factors in our nomogram can be easily obtained using preoperative abdominal imaging information and medical history. Additionally, we have conducted nomogram validations for patients undergoing different surgeries (the validation subjects being patients from the entire cohort). Supplementary figure represent subgroup validations for patients who simultaneously underwent LCBDE and either LC (A) or LH (B). Both subgroups showed high AUC values, indicating that the nomogram is applicable to patients undergoing the aforementioned surgeries.

In our cohort, 25 of the 41 patients who underwent conversion surgery because of dense abdominal adhesions had PUAS. PUAS can lead to extensive adhesions in the abdominal cavity and changes in the anatomical structure of the hepatic hilum. A previous autopsy confirmed that the incidence of abdominal adhesions after minor surgery, major surgery, and multiple abdominal surgeries were 51, 72, and 93%, respectively [18]. Wang et al. [1] found that the area with moderate to severe adhesions was very rich in vasculature, making the intraoperative surgical site anatomy difficult. Laparoscopic separation of adhesions is more likely to cause liver capsule injury and increase the risk of small intestine perforation, which makes the operation more complicated [19].

Tosun et al. [20] suggested that gallbladder wall thickening is the most sensitive indicator for conversion in LC. The thickening of the gallbladder wall often indicates the presence of acute or chronic inflammation. Chronic inflammation and/or acute inflammatory edema of the gallbladder often lead to an unclear anatomical relationship of the gallbladder triangle, or even “frozen” adhesion, making the operation more difficult. For patients with CBD stones, the occurrence of stone incarceration or repeated physical stimulation of stones to CBD wall easily causes inflammatory fibrosis of the CBD wall, resulting in local thickening [15]. In our study, we found that patients with localized thickening also had CBD edema during intraoperative exploration, resulting in brittleness of the CBD wall and its surrounding tissues. If laparoscopic exploration and suture are performed repeatedly at the edematous CBD, it easily leads to bleeding of the surrounding tissue of the bile duct during the operation and obstructs the operation field. Furthermore, it is also easy to cause postoperative bile leakage and increase the risk of complications [10].

In our nomogram, stone in the medial wall of the duodenum had a higher score (points = 86). Nobel et al. [21] showed that impaction of the medial wall of the duodenum stone was related to prolonged operative time and surgical failure in LCBDE. Because the lumen of the inner segment of the duodenum is small and the wall is thick, the stones in this area are easily impacted, and the removal basket and forceps for stone removal cannot penetrate this area along the wall of the bile duct or open it during the operation [12].

Xu et al. [10] found that large CBD stones were a risk factor for conversion to open surgery in LCBDE. However, there is no consensus on the definition of large CBD stones, and most researchers use a cut-off value between 10 and 15 mm [22, 23]. Sharma et al. [22] and Oguz [24] suggested using the ratio of the stone size/CBD size> 1 to define large stones. However, the diameter of the stone in the above studies included the transverse diameter and the long diameter; some stones were oval and had a long diameter even larger than the diameter of the CBD. In this case, the ratio could not be used to evaluate the size of the stone. Therefore, in our study, the maximum diameter of the stone was defined as the maximum transverse diameter perpendicular to the CBD wall. If the ratio is too large, it can cause stone impactionand increase the difficulty of allowing the stone removal basket and forceps through, thus causing difficulty in stone removal. Therefore, we suggest that appropriate conversion to laparotomy is necessary in the case of large stones or intramural stones to reduce the injury to the patient caused by repeated stone removal. Simultaneous LH has a high score in the nomogram. Surgery for patients with IHD stones is more challenging than those with liver neoplasm because IHD stones can lead to liver inflammation, which further leads to perihepatic adhesion and anatomical distortion [25]. Fibrosis and atrophic changes in the liver parenchyma lead to deformation of the anatomical structure of the IHD, making the transection of the liver parenchyma difficult [26].

As shown in Table 3, the operation time, postoperative antibiotic use time, and postoperative hospital stay time in the conversion group were longer than the patients in the non-conversion group, but there were no significant differences in stone clearance, mortality, and postoperative complication rates. This is consistent with previous studies [10, 16, 27]. Patients who should be converted if the surgeon insists on continuing laparoscopic exploration, the patients’ blood vessels, organs, and biliary tract may be seriously injured [11, 28, 29]. Therefore, it is necessary to convert to laparotomy in time when the treatment effect is similar.

We propose that laparoscopic surgery should be actively performed in low-risk patients. However, we recommend that additional surgical plans, such as ERCP + LC and open surgery, should be made before surgery for high-risk patients. We also recommend allocating low-risk patients to junior surgeons and high-risk patients to senior surgeons.

Our study had certain limitations. Firstly, this was a retrospective study based on the patients’ database. Selection bias cannot be completely ruled out, and prospective data are needed to further validate our prediction model in the future. Secondly, this was a single-center study, so the generalizability of our nomogram needs to be further validated in multicenter studies. We did not include variables like the type of PUAS, characteristics of hepatolithiasis, and the presence or absence of emergency surgery.

## Conclusion

Our study suggests seven independent risk factors for conversion to laparotomy in laparoscopic surgery for choledocholithiasis. We constructed a clinically effective and significant nomogram based on these factors. It can help surgeons evaluate the difficulty of surgery beforehand, optimize clinical decision-making, and provide a basis for timely conversion to open surgery.

### Supplementary Information

**Additional File 1.**

## Data Availability

The datasets used and/or analysed during the current study are available from the corresponding author on reasonable request.

## Abbreviations

CBD: Common bile duct
AUC: Area under the curve
DCA: Decision curve analysis
LCBDE: Laparoscopic common bile duct exploration
IHD: Intrahepatic bile duct
LH: Laparoscopic hepatectomy
BMI: Body mass index
PUAS: Previous upper abdominal surgery
ERCP: Endoscopic retrograde cholangiopancreatography
ALB: Albumin
LC: Laparoscopic cholecystectomy
ROC: Receiver operating characteristic
