# Supplementary figures and images for: A nomogram to predict conversion of laparoscopic surgery to laparotomy for Choledocholithiasis
Source: BMC Surg. 2023 Dec 8;23:372. doi: 10.1186/s12893-023-02275-1 (PMC10709908; doi:10.1186/s12893-023-02275-1)

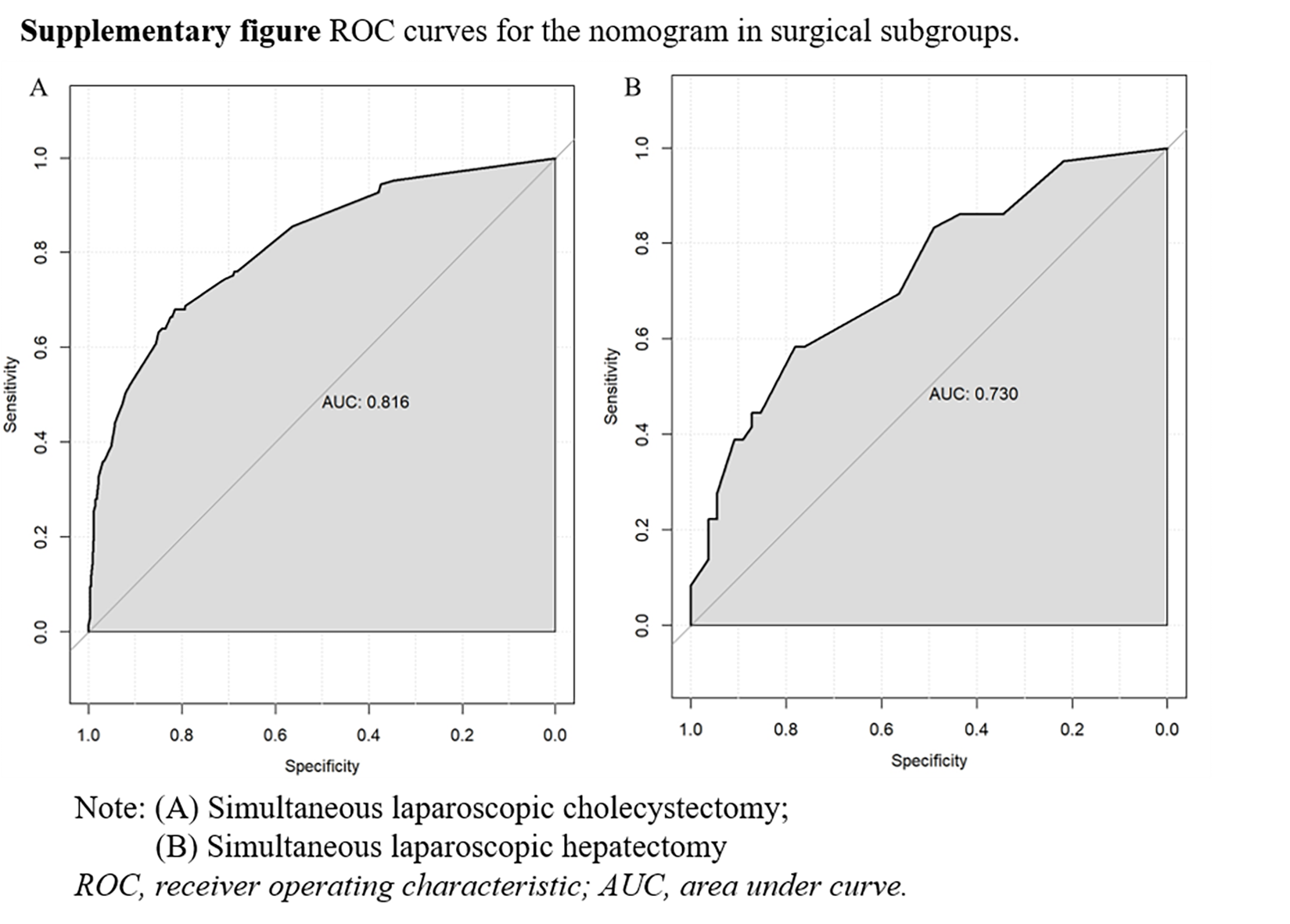

Supplement: Supplementary file 1 — Additional File 1. [file 12893_2023_2275_MOESM1_ESM.tif]
